# The Effectiveness and Safety of Bushen Huoxue Decoction on Treating Coronary Heart Disease: A Meta-Analysis

**DOI:** 10.1155/2021/5541228

**Published:** 2021-06-09

**Authors:** Lan-Chun Liu, Qi-Yuan Mao, Chao Liu, Jun Hu, Lian Duan, Jie Wang

**Affiliations:** ^1^Cardiovascular Department, Guang'anmen Hospital of China Academy of Chinese Medical Sciences, Beijing, China; ^2^Beijing University of Chinese Medicine, Beijing 100029, China; ^3^Department of Oncology, Guang'anmen Hospital of China Academy of Chinese Medical Sciences, Beijing, China

## Abstract

**Objective:**

The aim of this meta-analysis was to systematically evaluate the effectiveness and safety of the traditional Chinese medicine (TCM) formula Bushen Huoxue Decoction (BSHXD) in treating coronary heart disease (CHD).

**Methods:**

Randomized controlled trials (RCTS) of BSHXD in treating CHD were searched until March 2020, through six electronic databases: PubMed, Cochrane Library, CNKI, WanFang, SinoMed, and VIP. This study used the Cochrane Risk Test bias tool in the Cochrane Handbook to assess the quality of the methodology. Review Manager (RevMan) 5.3 was used to analyze the results. Grading of Recommendations Assessment, Development, and Evaluation (GRADE) criteria were applied in the classification of evidence quality.

**Results:**

Ten RCTs involving 901 patients were finally included in this meta-analysis. It revealed that the effectiveness of BSHXD in treating CHD was significantly better than that of the conventional western medicine (CWM) treatment (*P* < 0.00001). The effective rate of BSHXD treatment group on ECG was also significantly higher than that of CWM group (*P* < 0.00001). The low-density lipoprotein cholesterol was decreased in the treatment groups compared with those in the control groups (*P* < 0.00001). There was also a reduction in frequency and duration of angina pectoris (*P* < 0.00001). There were no significant differences in TC level (*P*=0.08), TG level (*P*=0.86), and HDL level (*P*=0.76) between the treatment and control groups. Five studies had informed adverse events, including nausea and diarrhea.

**Conclusion:**

Our findings laid the foundation to the use of TCM Formula BSHXD in combination with conventional western medicine for treating CHD. However, due to the limitation of the quality of the included researches, in addition to potential reporting bias, the above conclusions still need verification by higher-quality and better-designed studies.

## 1. Introduction

Coronary heart disease (CHD) is the representative of cardiovascular disease and the main cause of human death. According to the statistics of the World Health Organization, 17.9 million people died from cardiovascular disease worldwide [1]. At present, the number of patients with CHD in China has reached 11 million and the mortality rate has reached 113 per 100,000, which has continued to increase since 2012, causing a serious medical burden [2, 3].

CHD belongs to the category of “chest pain” and “heartache” in Chinese medicine. CHD is mostly based on blood stasis, cold coagulation, and phlegm turbidity, but the understanding of its syndrome elements should not be limited to those. In the previous study of our research team, it was found that Qi deficiency was the main pathogenesis through the investigation of 297 patients with CHD, of which the Kidney Qi deficiency was the main cause [4]. It shows that Kidney Qi plays an important role in maintaining the function of the heart. Then, our research team extracted the symptoms of 1069 patients with CHD. Among them, “fatigue, spontaneous sweating, dark purple tongue, and petechiae on tongue” were the main symptoms of Qi deficiency and blood stasis, accounting for about 21% [5].

With the research on the essence of kidney deficiency, modern scholars have expanded the theory of Bushen Huoxue Decoction (BSHXD). “Bushen” means tonifying kidney, “Huoxue” means promoting blood circulation. BSHXD is the general term for a series of traditional Chinese medicine formulas with the effect of tonifying the kidney and promoting blood circulation. A study found that patients with CHD treated with BSHXD had significant improvement in their angina pectoris symptoms, blood rheology, and blood lipid levels [6]. Many literatures have shown that kidney-tonifying drugs can improve blood rheology, inhibit platelet aggregation, and improve hematopoietic function. In addition, those who promote blood circulation can also improve kidney function, inhibit kidney thrombosis, and preserve potassium and diuresis. For example, *Rehmannia glutinosa polysaccharide*, the main chemical component of *Rehmannia glutinosa* (kidney-tonifying drug), can significantly improve hematopoietic function, especially on white blood cells and platelets [7]. *Cornus* (blood promoting drug) can tonify kidneys and also has the effect of promoting blood circulation. Experimental studies have shown that *Cornus* injection can significantly inhibit platelet aggregation and is positively correlated with its dosage [8]. Although the clinical efficacy of the BSHXD is commendable, it still lacks strong support from multicenter and large-sample clinical trials. Therefore, a meta-analysis is urgently needed to evaluate its effectiveness and safety in the treatment of CHD in order to provide a scientific basis for clinical decision making.

## 2. Materials and Methods

### 2.1. Study Registration

This study has been registered in PROSPERO (registration number: CRD42020173741). The review report is conducted in accordance with the preferred reporting items for systematic reviews and meta analyses (PRISMA) statement guidelines.

### 2.2. Search Strategy

We searched PubMed database, Cochrane Library, China National Knowledge Infrastructure (CNKI), WanFang Database, China Biomedical Document Service System (SinoMed), China Science and Technology Journal Full-Text Database (VIP) from its establishment to March 2020, including conference papers, dissertations, chapters in monographs, and other gray documents which may contain negative results. In the search strategy, this study chose the following terms as the subject terms (including title, abstract, keywords): “Coronary Artery Disease”, “Coronary Heart Disease”, “Activate Blood”, “Promote Blood”, “Kidney Nourish”, “Kidney Tonify”, “random allocation”, “*bu_shen*”, “*huo_xue*”, “*guan_xin_bing*”, “*xiong_bi*”, “*sui_ji*”, and “*dui_zhao*”. The search strategy is detailed in Table 1.

### 2.3. Inclusion Criteria

1. *Types of Patients.* There is no restriction on age, gender, or race; the included patients had been diagnosed with CHD through a clear definition or internationally recognized diagnostic criteria.
2. *Types of Interventions.* The intervention measure is BSHXD, which is mainly based on the theory of tonifying the kidney and promoting blood circulation. BSHXD is the only positive intervention in the treatment group compared with the control group. There are no restrictions on the dosage and duration of medication.
3. *Types of Comparison.* Conventional Western Medicine (CWM) treatment, such as isosorbide, nitroglycerin, and aspirin, can expand coronary artery, promote collateral circulation, promote blood redistribution, and increase myocardial oxygen supply. The specifications and dosage of CWM used in the control groups are the same as used in the treatment groups.
4. *Types of Outcomes.* The primary outcome included overall effective rate of angina pectoris. The secondary outcomes consisted of frequency and duration of angina pectoris, ECG effective rate, and blood lipid effective rate. All included literatures reported at least two results of the above.
5. *Types of Trials.* It is randomized controlled trial (RCT) using BSHXD combined with CWM to treat coronary heart disease. The language is Chinese or English.

### 2.4. Exclusion Criteria

1. Nonrandomized studies: although random is mentioned in the description, the entry method is the order of visits, case number, etc
2. Control group includes other methods of Chinese medicine: the setting of the control group is unreasonable, and acupuncture, Chinese patent medicine, or herbal extracts will not be included
3. Duplicate clinical data: repeated publication or repeated clinical data
4. Outcome effect is not clear: the data are incomplete, the outcome effect is not clear, the statistical method is incorrect, and the data cannot provide the mean and standard deviation

### 2.5. Data Extraction

Two researchers independently searched the database to screen the titles and abstracts of all search records to exclude irrelevant studies. The remaining studies were evaluated by reading the full text. Any disagreements were resolved through a third researcher. The researchers then extracted information from the literatures, including study ID, sample size, average age, sex ratio, CHD duration, diagnostic criteria, treatment group, control group, treatment duration, adverse event, and outcomes. We used predefined spreadsheets to extract data. For missing data or other detailed information, we will try to contact research investigators via e-mail for processing.

### 2.6. Quality Assessment

The researchers evaluated the quality of the included literature based on the Cochrane Handbook, including selection bias (random sequence generation and allocation hiding), implementation bias, measurement bias, follow-up bias, reporting bias, and other biases. According to the content of the literature, each indicator is judged by “low risk of bias”, “uncertain risk of bias”, and “high risk of bias”. We also used GRADEpro software in assessing the quality of evidence for each outcome in view of the GRADE approach. Finally, the evidence is classified into “high”, “moderate”, “low”, or “very low” quality, and the difference is resolved by a third party (Wang Jie).

### 2.7. Data Analysis

Statistical analysis was performed using RevMan 5.3 software. The continuous data use the mean difference (MD) or standardized mean difference (SMD) as the effect indicator, and the dichotomous data use the risk ratio (RR) as the effect indicator. Each effect size is given its point estimate and 95% confidence interval (CI). The Chi^2^ test was used for heterogeneity assessment. When the heterogeneity is significant (*P* < 0.10, *I*^*2*^ ≤ 50%), it can be considered that the included literature has homogeneity, and the fixed-effects model can be used for meta-analysis. Otherwise, it can be considered that the included literature is heterogeneous, and then we will further analyze the source of heterogeneity through sensitivity analysis and subgroup analysis. After excluding obvious clinical heterogeneity, a random effects model can be used for meta-analysis. Since the number of studies included in this meta-analysis is ≥ 10, a funnel chart can be used to test publication bias.

### 2.8. Outcomes

The main outcome indicator is the total effective rate of angina pectoris. The effective rate is determined according to the “Coronary Heart Disease Angina Pectoris and ECG Efficacy Evaluation Criteria” [9] or “Clinical Research Guidelines for Cardiovascular System Drugs” [10] or “Clinical Research Guidelines for New Chinese Medicines for the Treatment of Coronary Heart Disease” [11] or “Clinical Disease Diagnosis Based on Cure and Improvement Standards” [12] or related content in the “Standards for Emergency Diagnosis and Treatment of TCM Internal Medicine” [13]. It can be roughly divided into “Marked Effect” which represents angina pectoris basically disappeared, clinical symptoms are significantly improved, and nitroglycerin consumption is reduced by more than 80%; “Effective” which means angina attacks are reduced by 50% to 80%, clinical symptoms are improved, and nitroglycerin consumption is reduced by 50% to 80%; “Ineffective” which indicates no significant reduction in the number of angina pectoris, no significant improvement in clinical symptoms, less than 50% reduction in nitroglycerin consumption; “exacerbation” which suggests more angina pectoris, clinical symptoms aggravated, and nitroglycerin consumption increased. The secondary outcomes consist of frequency and duration of angina pectoris, ECG effective rate, total cholesterol (TC), triglycerides (TG), high density lipoprotein (HDL), and low-density lipoprotein (LDL).

## 3. Results

### 3.1. Literatures Search and Selection

**Figure 1:**
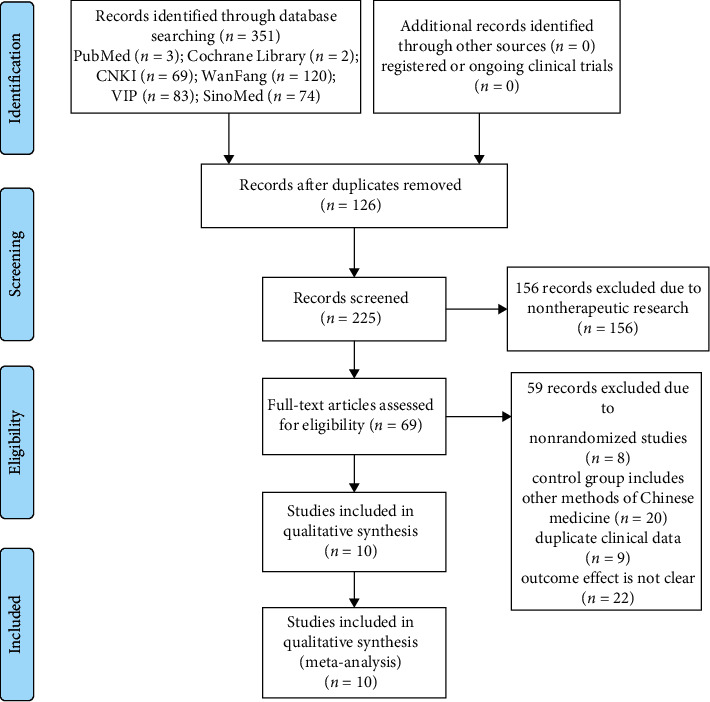
Flowchart of the literature retrieval.

A preliminary search of 351 related articles was conducted, including 3 articles in PubMed database, 2 articles in Cochrane Library database, 69 articles in China National Knowledge Infrastructure (CNKI), 120 articles in WanFang database, 74 articles in China Biomedical Document Service System (SinoMed), and 83 articles in China Science and Technology Journal Full-Text Database (VIP). The document management software NoteExpress was used to eliminate 126 duplicate studies, and 225 documents remained after deduplication. 156 nontherapeutic articles were deleted for the preliminary screening of the remaining literature reading titles and abstracts, and the remaining 69 articles were rescreened. After rescreening the full text, 59 articles were excluded, including 8 nonrandomized controlled trials, 20 control groups did not meet the standards, 9 clinical data were duplicated, and 22 outcome indicators or baseline data were missing. After collating the data, the final number of documents included in the qualitative analysis is 10, including a total of 901 patients. The screening process is shown in Figure 1 and follows the PRISMA principle [14].

### 3.2. Characteristics of the Included Literatures

Characteristics of the 10 studies are shown in Table 1. All included studies came out between 2005 and 2017. The 10 RCTs involved 901 patients, including 457 in treatment groups and 444 in control groups. The sample size for each study ranged from 50 to 189. All patients included in the studies met the diagnostic criteria for CHD. Seven studies used the criteria of the World Health Organization Task Force on Standardization of Clinical Nomenclature (WHO1979) [15]. One study referred to the Department of Pharmaceutical Administration Guidelines for clinical research on cardiovascular system drugs (DPA1998) [10]. Another used the Detection and Risk Assessment of stable ischemic heart disease (DRA2014) [16]. The remaining study was based on the Chinese Journal of Cardiovascular Diseases Guidelines for the diagnosis and treatment of chronic stable angina pectoris (CJCD2007) [17]. The characteristics of the included literatures are shown in Table 2.

### 3.3. Risk of Bias in the Included Literatures

**Figure 2:**
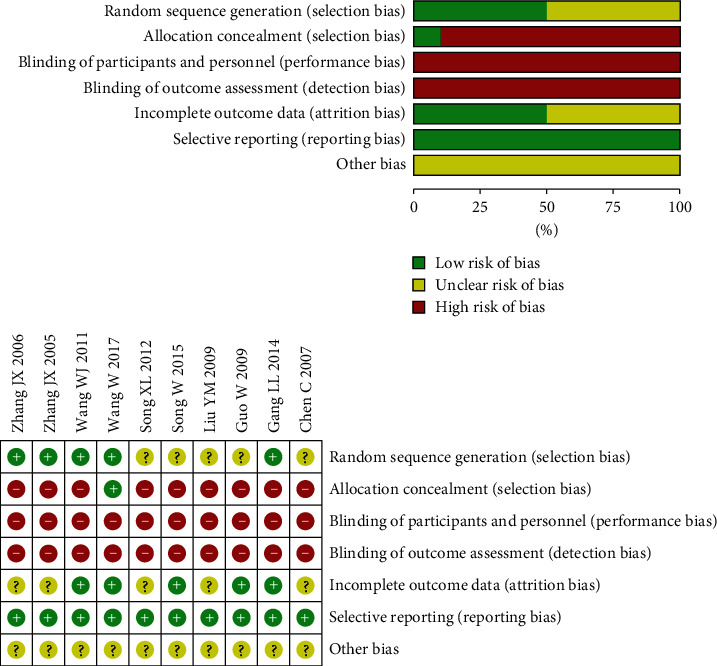
Risk and bias graph.

The baseline conditions of the included 10 articles [18–27] were basically consistent and comparable. All studies mentioned randomization, but 5 of them [20–22, 24, 26] did not specify the randomization method. Only one study [27] assigned patient numbers and assigned them according to their corresponding digital numbers, and it was difficult to achieve strict allocation concealment in the remaining trials. All the control groups included in the study did not use placebo drugs, so the patients and the investigators responsible for recruitment could not be well blinded, and the measurement of outcome indicators might be affected by the lack of blinding. Therefore, the implementation bias and measurement bias of the 10 studies are all “high risk”. Five studies [21, 23, 25–27] reported on safety indicators while the remaining five did not mention or follow-up information was not enough to determine the risk level. All predeclared outcomes in the 10 articles have been reported, but there is no enough information to evaluate whether there are other risks of bias.  Figure 2 depicted the quality evaluation of the included literatures.

### 3.4. Primary Outcome

**Figure 3:**
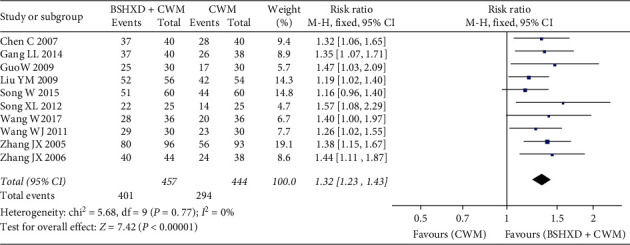
Forest plots of BSHXD on overall effective rate.

The primary outcome indicator is the total effective rate of angina pectoris which had been reported in ten studies [18–27], and the results significantly showed differences between two groups. The fixed-effects model was performed for statistical analysis, because these trials exhibited nonsignificant heterogeneity (*χ*^2^ = 5.68, *P*=0.77, *I*^2^ = 0％). The effective rate of the treatment group combined with BSHXD was significantly higher than that of the control group [*N* = 901, RR 1.32, 95% CI (1.23, 1.43), *Z* = 7.42, *P* < 0.00001] (Figure 3).

### 3.5. Secondary Outcome

#### 3.5.1. Frequency and Duration of Angina Pectoris

**Figure 4:**
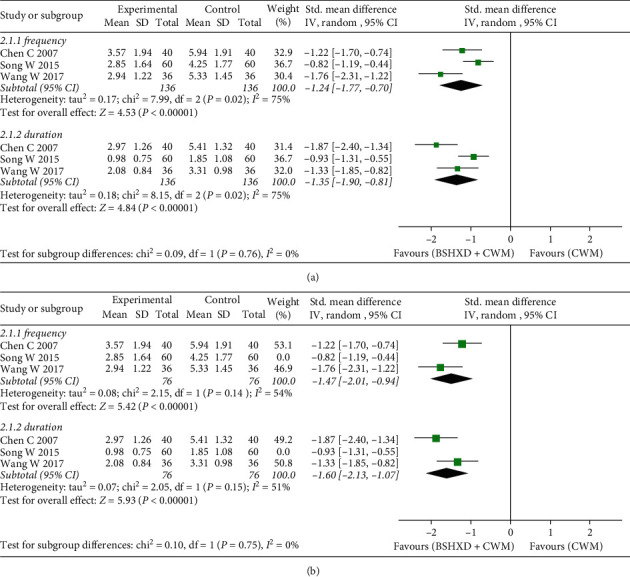
Forest plots of BSHXD on frequency and duration of angina pectoris. (a) The effect of BSHXD on angina pectoris. (b) Sensitivity analysis of the effect of BSHXD on angina pectoris.

Three studies reported the frequency and duration of angina pectoris with a total of 272 patients [20, 26, 27]. The results indicated that the frequency of angina pectoris in the treatment group with BSHXD was lower than that in the control group [SMD =-−1.24, 95% CI (−1.77,-0.70), *Z* = 4.53, *P* < 0.00001]. Due to the significant heterogeneity in the study (*χ*^2^ = 7.99, *P*=0.02, *I*^2^ = 75%), a random effects model was used. The duration of angina pectoris in the treatment group with BSHXD was shorter than that in the control group [SMD = −1.35, 95% CI (−1.90, −0.81), *Z* = 4.84, *P* < 0.00001], but the results showed severe heterogeneity (*χ*^2^ = 8.15, *P*=0.02, *I*^2^ = 75%); therefore, the random effects model was adopted for meta-analysis (Figure 4(a)). We used sensitivity analysis to evaluate the source of heterogeneity and found that after removing Song's study [26], the heterogeneity of angina pectoris episodes was significantly reduced, and it can be considered that this study may be the source of heterogeneity (Figure 4(b)).

#### 3.5.2. ECG Efficiency

**Figure 5:**
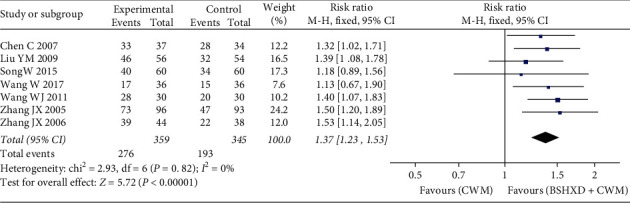
Forest plots of BSHXD on ECG efficiency.

The ECG efficiency analysis included a total of 704 patients who were included in seven studies [18–20, 22, 23, 26, 27]. The results of meta-analysis showed that the effective rate of the treatment group with BSHXD was significantly higher than that of the control group [RR = 1.37, 95% CI (1.23, 1.53), *Z* = 5.72, *P* < 0.00001], and the difference is statistically significant. The homogeneity of these included studies is not significant (*χ*^2^ = 2.93, *P*=0.82, *I*^2^ = 0%), so the fixed-effects model was conducted for statistical analysis (Figure 5).

#### 3.5.3. Lipid Levels

**Figure 6:**
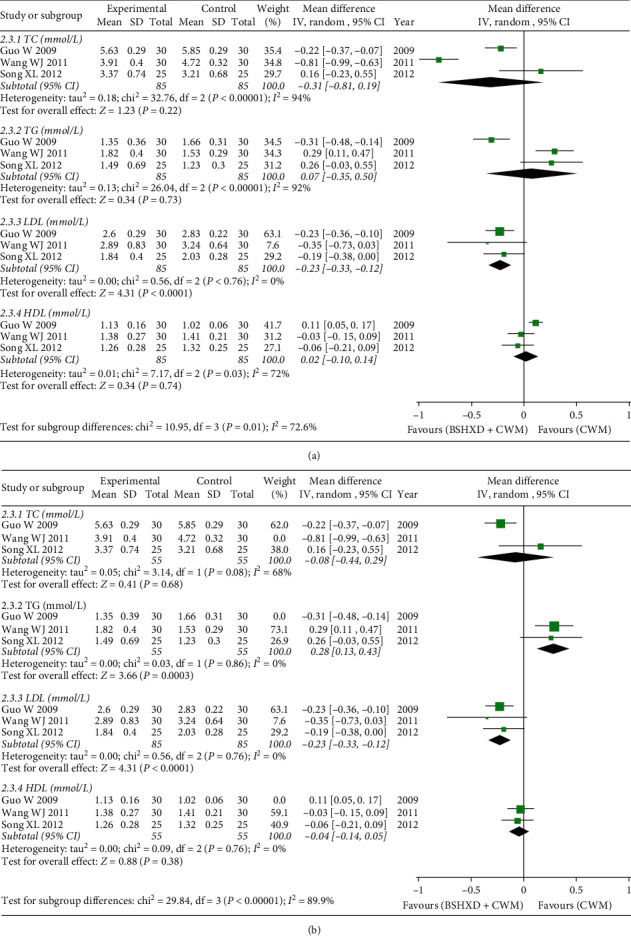
Forest plots of BSHXD on lipid levels. (a) The effect of BSHXD on lipid levels. (b) Sensitivity analysis of the effect of BSHXD on lipid levels.

Three studies reported on blood lipid levels [21, 23, 24], which included four items: total cholesterol (TC), triglycerides (TG), high density lipoprotein (HDL), and low-density lipoprotein (LDL). The results of the meta-analysis showed that BSHXD has the effect of reducing LDL levels [MD = −0.23, 95% CI (−0.33, −0.12), *Z* = 4.31, *P* < 0.00001]. On the contrary, regarding the effect of BSHXD on TC, TG, and HDL levels, there was no significant difference between the treatment group and the control group, and the 95% confidence interval (95% CI) crossed the invalid line (Figure 6(a)). By exploring the reasons for the heterogeneity through sensitivity analysis, we found that after removing Wang WJ's [23] research the heterogeneity of TC can be reduced (*χ*^2^ = 3.14, *P*=0.08, *I*^2^ = 68%), but its confidence interval still intersects the invalid line. In the same way, by removing the research of Guo W [21] the heterogeneity of TG (*χ*^2^ = 0.03, *P*=0.86, *I*^2^ = 0%) and HDL (*χ*^2^ = 0.09, *P*=0.76, *I*^2^ = 0%) can be greatly reduced. At this time, the 95% CI of TG level is far from the invalid line, and the baseline TG level of the remaining two studies is low, which may indicate that the BSHXD has a better effect on patients with lower baseline TG level [MD = 0.28, 95% CI (0.13, 0.43), *Z* = 3.66, *P*=0.0003], and the difference is statistically significant (Figure 6(b)).

#### 3.5.4. Adverse Events

A total of five studies [21, 23, 25–27] published adverse reactions and safety indicators, among which there were 2 cases of nausea in the treatment group and 1 case of diarrhea in the control group [25], but it has not been proven to be related to the effect of the BSHXD. Meta-analysis was not carried out due to the limitation of adverse reactions reports.

#### 3.5.5. Publication Bias

**Figure 7:**
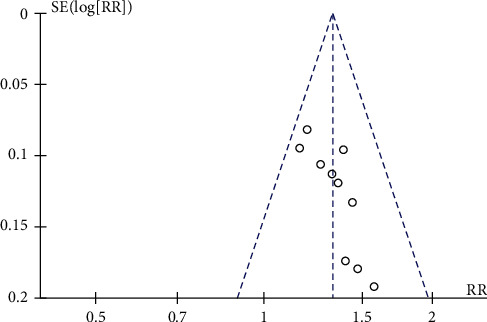
Funnel plots of BSHXD on overall effective rate.

A funnel chart analysis of the main outcome index of angina pectoris [18–27] found that all studies were not arranged symmetrically around the center line of the funnel chart, and the funnel chart had missing corners, indicating a certain publication bias. It suggests that there are probably some negative results (Figure 7).

### 3.6. GRADE Assessment

According to GRADE assessment, the quality evidence of the main outcome indicators was considered to be moderate evidence, and the secondary outcomes were rated as moderate, low, or very low as shown in Table 3.

## 4. Discussion

Traditional Chinese medicine believes that heart and kidney are intertwined, and kidney deficiency may be the fundamental cause of coronary heart disease. Therefore, when using traditional Chinese medicine to treat CHD, it is customary to add herbal medicine for tonifying the kidney which is as important as promoting blood circulation. Tonifying the kidney takes radical measures, which can fill the essence and strengthen the body deficiency. Promoting blood circulation takes stopgap measures, which can cure its symptoms by promoting blood circulation and removing blood stasis. Conventional antianginal therapies (such as isosorbide, nitroglycerin, and aspirin) can dilate the coronary arteries, promote collateral circulation, redistribute blood, and increase the oxygen supply to the heart muscle. BSHXD is composed of a series of traditional Chinese medicines that have the effect of nourishing the kidney and promoting blood circulation. The meta-analysis indicated that its curative effect on coronary heart disease and angina pectoris is better than the CWM treatment which promotes blood circulation alone.

The meta-analysis indicates that BSHXD for CHD was superior to that of the CWM group in terms of total effective rate of angina pectoris. Comparing with CWM treatment group, the combination of BSHXD led to a mean greater reduction in frequency and duration of angina pectoris, with statistically significant between-study heterogeneity. Further reading and evaluation found that the TCM syndrome type included in the study that produced heterogeneity [26] was the syndrome of heart-kidney yin deficiency, while the other two studies were not limited to this type of TCM syndrome. It was suggested that the effect of BSHXD on frequency and duration of angina pectoris may be closely related to the TCM syndrome type. The effective rate of BSHXD treatment group on ECG curative effect was also significantly higher than that of CWM group.

Additionally, BSHXD exhibited advantages in improving blood lipid LDL level in CHD patients, but not in lowering TC, TG, and HDL level and had great heterogeneity. Through sensitivity analysis, it was found that the baseline blood lipid level before treatment might be the cause of the heterogeneity. By removing the study of Wang WJ [23], the heterogeneity of TC can be greatly reduced (*I*^*2*^ = 68%). Further reading and evaluation found that the baseline TC level before treatment from high to low was Guo W 2009 > Wang WJ 2011 > Song XL 2012, but the 95% confidence interval of the remaining studies [21, 24] still intersected the invalid line, which suggested that although the heterogeneity is reduced (*I*^*2*^ from 94% to 68%), patients with a baseline TC level that is too high or too low are not sensitive to BSHXD. That is, BSHXD may not significantly improve the level of TC. Sensitivity analysis found that removing the study of Guo W [21] could greatly reduce the heterogeneity of TG and HDL (*I*^*2*^ = 0%), and the remaining studies [23, 24] had lower TG level and the difference was statistically significant, which suggested that BSHXD might have a better effectiveness on patients with lower baseline of TG levels. Similarly, it was found that the HDL level of the remaining studies [23, 24] was higher, but the 95% confidence interval still intersected the invalid line, suggesting that the effectiveness of BSHXD on patients with high baseline HDL levels might not be different from CWM treatment. In summary, the sensitivity analysis results suggested that when comparing the effectiveness of BSHXD on blood lipid levels, we need to consider the baseline level, conduct a stratified analysis, or add interaction items for adjustment.

Regrettably, the included studies did not report kidney function indicators as outcomes. There is a lack of research for BSHXD in the treatment of CHD. Future research ideas should focus on observing the improvement of kidney function while still paying attention to the improvement of heart function. Epidemiological investigations have reported more and more reports on the relationship between early chronic kidney disease (CKD) and CHD. For example, it was found that in patients after myocardial infarction, microalbuminuria can appear within a few hours and is proportional to the area of the infarction [28]. Microalbuminuria is related to the inflammatory process, and the status of inflammation in the CHD has been confirmed by a large number of clinical experiments [29]. In the Multiple Risk Factor Intervention Trial (MRFIT) study, it was found that elevated serum creatinine (Cr) concentration was probably an independent risk factor for death from CHD [30]. In the Hypertension Detection and Follow-up Program (HDFP) study, it showed that CKD patients' mortality rate due to cardiovascular disease was much higher than that due to Cr ≥ 1.7 mg/dl, of which 58% died of cardiovascular disease and only 19% died of kidney failure [31]. In the Heart Outcomes and Prevention Evaluation study (HOPE) experiment, it was found that that patients with mild chronic renal insufficiency [Cr in the range of 1.4 mg/dl to 2.3 mg/dl] have a significantly increased incidence of myocardial infarction [32]. After summarizing the most valuable studies, the American Heart Association (AHA) also believed that CKD is a risk factor for cardiogenic death [33]. In the GUSTO study, the results of multivariate analysis concluded that, compared with other risk factors such as stroke, heart failure, elevated C-reactive protein, and ST-segment changes, CKD is more related to death and myocardial infarction [34]. Framingham Study found that only 10 of the 198 patients with CKD who died have developed renal failure, and most of the patients died of cardiovascular disease [35]. The latest clinical application guidelines of K/DOQ1 also pointed out that most patients with CKD died before they developed renal failure. Among them, cardiovascular diseases (including CHD, heart failure, and cerebrovascular disease) are the main cause of death. The guidelines believe that all patients with chronic kidney disease should be regarded as high-risk groups of cardiovascular disease [36].

## 5. Conclusions

The main purpose of this meta-analysis is to evaluate RCTs of Bushen Huoxue Decoction in the treatment of coronary heart disease through comprehensive retrieval. It suggested that BSHXD was superior to that of the CWM treatment in the improvement of overall effective rate of angina pectoris, reduction of frequency and duration of angina pectoris, and lowered LDL level. CHD patients, especially who have a low TG baseline level, might be sensitive with BSHXD combined with conventional western medicine. However, BSHXD may not be the best choice to improve abnormal indexes of TC and HDL in CHD patients.

However, studies on the adverse reactions of BSHXD in the treatment of CHD are very limited, so no quantitative meta-analysis had been conducted for it yet. The funnel chart results indicated that despite extensive searches, potential publication bias could not be ruled out. The follow-up time included in the literatures was relatively short, and long-term studies are still needed to further verify the results. Some of the outcome indicators included in the study had obvious heterogeneity. Sensitivity analysis suggested the heterogeneity might be related to the baseline level of the indicator. Therefore, further studies such as multicenter and large-scale RCTs are needed to build the most suitable method for clinical treatment and management of patients with coronary heart disease.

## Figures and Tables

**Table 1:** Search strategy.

Database	Items	Number	Search items
*PubMed*	3	#1	(((((((“Bushen”[All Fields] OR “Huoxue”[All Fields]) OR “Activate Blood “[All Fields]) OR “Promote Blood Circulation”[All Fields]) OR “Eliminate Stasis”[All Fields]) OR “ Remove Stasis”[All Fields]) OR “ Disperse Blood Stasis”[All Fields]) OR “ xue yu”[All Fields]) OR “ huo xue hua yu”[All Fields]) OR “Kidney Tonify”[All Fields]) OR “Kidney Nourish”[All Fields])
		#2	(((((((“Coronary Artery Disease”[MeSH Terms] OR “Coronary Artery Disease”[Title/Abstract]) OR “Coronary Heart Disease “[Title/Abstract]) OR “Coronary Arterioscleroses”[Title/Abstract]) OR “Coronary Atheroscleroses”[Title/Abstract]) OR “ Coronary Atherosclerosis”[Title/Abstract]) OR “Chest Blocking”[Title/Abstract]) OR “chest obstruction”[Title/Abstract])
		#3	((“random allocation”[MeSH Terms] OR (“random”[All Fields] AND “allocation”[All Fields]) OR “random allocation”[All Fields] OR “randomized”[All Fields]) OR (“clinical trials as topic”[MeSH Terms] OR (“clinical”[All Fields] AND “trials”[All Fields] AND “topic”[All Fields]) OR “clinical trials as topic”[All Fields] OR “trial”[All Fields]))
		#4	Numbers 1–3

*Cochrane Library*	2	#1	MeSH descriptor: [Coronary Artery Disease] explode all trees
		#2	(Coronary Heart Disease): ti, ab, kw (Word variations have been searched)
		#3	(Chest Blocking): ti,ab,kw (Word variations have been searched)
		#4	(chest obstruction): ti,ab,kw (Word variations have been searched)
		#5	#1 OR #2 OR #3 OR #4
		#6	(bushen): ti,ab,kw
		#7	(Kidney Tonify): ti,ab,kw (Word variations have been searched)
		#8	(Kidney Nourish): ti,ab,kw (Word variations have been searched)
		#9	#6 OR #7 OR #8
		#10	(huoxue): ti,ab,kw
		#11	(Activate Blood): ti,ab,kw (Word variations have been searched)
		#12	(Promote Blood Circulation): ti,ab,kw
		#13	(Remove Stasis): ti,ab,kw (Word variations have been searched)
		#14	#11 OR #12 OR #13
		#15	#9 AND #14
		#16	#15AND #5

CNKI	69	#1	SU =( *'bu_shen'*+ *'yi_shen'* +*'gu_shen' *) ∗( *'huo_xue'* + *'hua_yu'* +*'qu_yu'* ) ∗( *'guan_xin_bing'* + *'xiong_bi'*) ∗( *'sui_ji'*+ *'dui_zhao'*)
WanFang	120	#1	subject: (*'bu_shen'*+*'yi_shen'*+*'gu_shen'*)∗subject: (*'huo_xue'*+*'hua_yu'*+*'qu_yu'*)∗subject: (*'guan_xin_bing'*+*'xiong_bi'*)∗subject: (*'sui_ji' *∗ *'dui_zhao'*)

SinoMed	74	#1	*'sui_ji' *[common fields] AND *′'dui_zhao' *[common fields]) AND (*'guan_xin_bing' *[common fields] OR *'xiong_bi'*[common fields]) AND (*'huo_xue' *[common fields] OR *′'hua_yu'* [common fields] OR *′'qu_yu' *[common fields]) AND (*'bu_shen' *[common fields] OR *′'yi_shen' *[common fields] OR*'gu_shen' *[common fields]

VIP	83	#1	Any field=*'bu_shen'* AND Any field=*'huo_xue'* AND Any field=*'guan_xin_bing'*

**Table 2:** Characteristics of the included literatures.

Study ID	Sample size (T/C)	Average age (T)	Average age (C)	Sex (M/F)	CHD duration (years)	Diagnostic criteria	Intervention (T)	Intervention (C)	Treatment duration (days)	Adverse event	Outcomes
Zhang, 2005 [18]	96/93	56.3	58.5	T: 57/39 C: 56/37	T: 2.4 C: 2.8	WHO1979	C + BSHXD	Nitrate, *β*RB, Aspirin	7	Not available	①③
Zhang, 2006 [19]	44/38	57.3	56.6	T: 23/21 C: 21/17	T: 3.7 C: 3.6	DPA1998	C + BSHXD	Nitrate, *β*RB, Aspirin	90	Not available	①③
Chen and Chen, 2007 [20]	40/40	61.73 ± 6.54	60.94 ± 6.72	T: 24/16 C: 23/17	T: 9.84 ± 3.43 C: 9.46 ± 3.51	WHO1979	C + BSHXD	Nitrate, Aspirin	30	Not available	①②③
Guo, 2009 [21]	30/30	65.5 ± 6.87	67.7 ± 7.32	T: 16/14 C: 13/17	—	WHO1979	C + BSHXD	Aspirin, Clopidogrel	21	Restenosis	①④
Liu, 2009 [22]	56/54	47–58	47–58	T: 0/56 C: 0/54	—	WHO1979	C + BSHXD	Nitrate, *β*RB, Aspirin, Statins	180	Not available	①③
Wang, 2011 [23]	30/30	57.10 ± 7.62	58.06 ± 8.40	T: 14/16 C: 13/17	—	WHO1979	C + BSHXD	Nitrate, *β*RB, Aspirin, Statins	28	Not found	①③④
Song and Shi, 2012 [24]	25/25	67.36 ± 6.49	67.84 ± 5.28	T: 15/10 C: 13/12	T: 9.02 ± 10.89 C: 8.35 ± 11.25	WHO1979	C + BSHXD	Nitrate, *β*RB, Aspirin, Statins	28	Not available	①④
Gang et al., 2014 [25]	40/38	63.5 ± 6.8	63.0 ± 6.2	T: 20/20 C: 20/18	T: 5.18 ± 1.4 C: 5.7 ± 1.4	DRA2014	C + BSHXD	Nitrate, *β*RB, Aspirin, Clopidogrel, Statins	14	Gastrointestinal reaction	①
Song et al., 2015 [26]	60/60	55.31 ± 8.65	56.63 ± 7.85	T: 29/31 C: 32/28	T: 1–13 C: 2–14	CJCD2007	C + BSHXD	Nitrate, *β*RB, Aspirin	28	Not found	①②③
Wang, 2017 [27]	36/36	35–75	35–75	T: 20/16 C: 19/17	—	WHO1979	C + BSHXD	Aspirin, Clopidogrel, Statins	21	Not found	①②③

①: total effective rate, ②: attack of angina pectoris, ③: improvement of ECG, and ④: serum biochemical indicators. BSHXD: Bushen Huoxue Decoction; CWM: conventional western medicine; T: treatment group; C: control group; M/F: men/female; CHD: coronary heart disease; WHO1979: World Health Organization Task Force on Standardization of Clinical Nomenclature; DPA1998: Department of Pharmaceutical Administration Guidelines for clinical research on cardiovascular system drugs; DRA2014: Detection and Risk Assessment of stable ischemic heart disease; CJCD2007: Chinese Journal of Cardiovascular Diseases Guidelines for the diagnosis and treatment of chronic stable angina pectoris.

**Table 3:** GRADE assessment of quality of the outcome indicators.

Quality assessment							No. of patients		Effect		Quality	Importance
No. of studies	Design	Risk of bias	Inconsistency	Indirectness	Imprecision	Other considerations	BSHXD	Control	Relative (95% cl)	Absolute		
*Total effective rate*												
10	Randomized trials	Serious^1^	No serious inconsistency	No serious indirectness	No serious imprecision	None	401/457 (87.7%)	294/444 (66.2%)	RR 1.32 (1.23 to 1.43)	212 more per 1000 (from 152 more to 285 more)	⊕⊕⊕ooo MODERATE	CRITICAL
								65.8%		211 more per 1000 (from 151 more to 283 more)		

*Attack of angina pectoris-frequency (better indicated by lower values)*												
3	Randomized trials	Serious^2^	Serious^3^	No serious indirectness	No serious imprecision	None	136	136	—	SMD1.24 lower (1.77 to 0.7 lower)	⊕⊕oo LOW	IMPORTANT

*Attack of angina pectoris-duration (better indicated by lower values)*												
3	Randomized trials	Serious^2^	Very serious^4^	No serious indirectness	No serious imprecision	None	136	136	—	SMD1.35 lower (1.9 to 0.81 lower)	⊕ooo VERY LOW	IMPORTANT

*Improvement of ECG*												
7	Randomized trials	Serious^2^	No serious inconsistency	No serious indirectness	No serious imprecision	None	276/359 (76.9%)	193/345 (55.9%)	RR1.37 (1.23 to 1.53)	207 more per 1000 (from 129 more to 296 more)	⊕⊕⊕o MODERATE	IMPORTANT
								57.9%		214 more per 1000 (from 133 more to 307 more)		

*Serum biochemical indicators-TC (mmol/L) (better indicated by lower values)*												
2	Randomized trials	Serious^2^	Very serious^5^	No serious indirectness	Serious^6^	None	85	85	—	MD 0.08 lower (0.44 lower to 0.29 higher)	⊕ooo VERY LOW	NOT IMPORTANT

*Serum biochemical indicators-TG (mmol/L) (Better indicated by lower values)*												
2	Randomized trials	Serious^2^	Very serious^7^	No serious indirectness	Serious^6^	None	85	85	—	MD 0.28 higher (0.13 to 0.43 higher)	⊕ooo VERY LOW	NOT IMPORTANT

*Serum biochemical indicators・LDL (mmol/L) (better indicated by lower values)*												
3	Randomized trials	Serious^2^	No serious inconsistency	No serious indirectness	No serious imprecision	None	85	85	—	MD 0.23 lower (0.33 to 0.12 lower)	⊕⊕⊕o MODERATE	IMPORTANT

*Serum biochemical indicators-HDL (mmol/L) (better indicated by lower values)*												
2	Randomized trials	Serious^2^	Serious^8^	No serious indirectness	Serious^6^	None	85	85	—	MD 0.04 lower (0.14 lower to 0.05 higher)	⊕ooo VERY LOW	NOT IMPORTANT

^1^Blind method of the included study was not mentioned; ^2^blind method of the included study was not mentioned; ^3^significant heterogeneity, *I*^2^ = 67%; ^4^significant heterogeneity, *I*^2^ = 91%; ^5^significant heterogeneity, *I*^2^ = 94%; ^6^ the 95% Cl crosses the invalid line; ^7^significant heterogeneity, *I*^2^ = 92%; ^8^significant heterogeneity, *I*^2^ = 72%.

## Data Availability

All the data generated or analyzed during the study are available and included in this published article.

## Supplementary Materials

Supplementary Materials The supplementary file contains the PRISMA 2009 checklist.
